# Immune cell landscape analysis reveals prognostic immune cells and its potential mechanism in squamous cell lung carcinoma

**DOI:** 10.7717/peerj.9996

**Published:** 2020-10-05

**Authors:** Yongyong Wang, Jianji Guo

**Affiliations:** Cardio-Thoracic Surgery, First Affiliated Hospital of Guangxi Medical University, Nanning, China

**Keywords:** Squamous cell lung carcinoma, T follicular helper cells, PWRN1, ceRNA, Overall survival

## Abstract

**Background:**

Squamous cell lung carcinoma (LUSC) was closely associated with smoking which was known to have a distant immunosuppression effect. In this study, we aimed to explore the relationship between immune cells and clinical outcomes of LUSC patients with smoking history.

**Methods:**

The immune cell infiltration and RNA expression profiles of LUSC patients were collected from The Cancer Genome Atlas (TCGA). Then, the correlation between immune cell infiltration and clinical characteristics was explored. According to the level of immune cell infiltration, LUSC patients with smoking history were divided into high or low group to screen the differentially expressed lncRNAs and mRNAs. The prediction of target genes was performed by miRanda. Finally, the prognostic value of a certain signature was confirmed in an independent dataset.

**Results:**

Higher abundance of tumor-infiltrating T follicular helper (Tfh) cells together with a lower abundance of resting memory CD4 T cells had been found in LUSC current reformed smokers for ≤15 years and current smoking patients. Moreover, Tfh cell infiltration was not only associated with better overall survival (OS) but also varied from different degrees of TNM stage. Low expression of lncRNA PWRN1 and its potential regulating genes DMRTB1, PIRT, APOBEC1, and ZPBP2 were associated with better OS. Combining PWRN1 and four regulating genes as a signature, patients with higher-level expression of the signature had shorter survival time in not only the TCGA but also in the GEO dataset.

**Conclusions:**

It was found that Tfh cells presented higher infiltration in LUSC current reformed smokers for ≤15 years and current smokers, while resting memory CD4 T cells had lower infiltration. The signature consisting of PWRN1 as well as its predicted targeted mRNAs was dysregulated in different levels of Tfh cell infiltration and might indicate patients’ OS.

## Introduction

Since 2010, lung cancer has been the leading cause of cancer-related death worldwide (Ding et al., 2019). Non-small cell lung cancer (NSCLC), the most common kind of lung cancer (accounting for 83% of total lung cancer cases), has a poor 5-year survival rate, which is lower than 20%, although great efforts and progresses have been made in chemotherapy, radiotherapy, surgery and immunotherapy (Miller et al., 2019; Wang et al., 2020).

For decades, the risk of lung cancer from smoking has been well established through epidemiological methods (Alberg et al., 2007; Okamoto et al., 2014). Squamous cell lung carcinoma (LUSC), a subtype of NSCLC, had a tight association with tobacco smoking as 96% of LUSC cases had smoking history (Cancer Genome Atlas Research, 2012; Kenfield et al., 2008; Khuder & Mutgi, 2001). Tobacco smoking promotes cancer progression mainly in two ways. Firstly, cigarette smoking is tumorigenic by causing gene alterations, such as the major tumor suppressor gene TP53, despite only a small proportion of smokers eventually developed into cancer (Alberg, Brock & Samet, 2005). Secondly, it could influence defense mechanism by suppressing NK cell number and activity as well as cytotoxic T cell activity (Chalmer, Holt & Keast, 1975; Mio et al., 1997; Ng et al., 2006; Sopori, 2002; Stampfli & Anderson, 2009; Swann et al., 2007; Woodruff et al., 2005), being a negative prognostic factor for cancer patients. However, few studies have learned about the relationship among changes in smoking status, mortality, and immune cell infiltration, which was focused in our research.

The T follicular helper (Tfh) cell, a novel type of CD4+ T helper cell that has been discovered recently, interacts closely with B cells resulting in B cell proliferation and activation (Ramiscal & Vinuesa, 2013). The effect of tobacco smoking on Tfh cells infiltration in LUSC patients has not been studied thoroughly in the past investigations. Previous studies found that Tfh cell was positively associated with OS of NSCLC (Ma et al., 2016). However, the underlying mechanism still need further exploration and in this study, we tried to explain it by the dysregulated long non-coding RNA (lncRNA)-mRNA pairs in patients with different degrees of immune cell infiltration. That has not been reported before, so present research may offer some novel points to investigate Tfh cells.

LncRNAs, transcripts longer than 200 bp without protein-coding potential, have been found to play an important role in tumor formation and development (Heery et al., 2017; Lin & Yang, 2018). LncRNAs could regulate the expression of mRNA by competing with shared miRNAs, which was defined as competing endogenous RNAs (ceRNAs) (Li et al., 2019). CeRNA network had been investigated in multiply researches to probe prognosis biomarker of cancer or explain molecular processes during tumorigenesis and metastasis (Cheng et al., 2019; Li et al., 2020). Combined with OS and differentially expressed analysis, the related research of lncRNAs will also help to reveal some novel prognosis biomarkers.

In this study, the immune cell infiltration data and RNA expression profiles of LUSC patients were collected from The Cancer Genome Atlas (TCGA). Then, the correlation of immune cell infiltration with patient clinical characteristics such as gender, TNM stage, and smoking history, were explored. According to the different abundance of immune cell infiltration that presented in reformed smokers, current smokers, and non-smoking patients, LUSC patients with smoking history were divided into high and low infiltration group. The differentially expressed lncRNAs and mRNAs were screened to construct ceRNA network so that we can further find explanations of immune cell in prognosis evaluation and potential prognosis biomarkers. Finally, the prognostic value of certain signature was confirmed in another dataset downloaded from GEO.

## Materials and Methods

### Data collection

Firstly, the immune cell fraction and survival data of 490 LUSC patients from TCGA were downloaded, which had been reported by Thorsson et al. (2018). The relative fraction of 22 immune cell types of each patient was estimated using CIBERSORT (Newman et al., 2015) based on TCGA RNA-seq data. CIBERSORT is a computational method that can accurately calculate relative levels of various immune cell types in complex gene expression mixtures. Using gene expression signatures of 547 genes (LM22 files) as an input matrix, CIBERSORT can characterize and quantify immune cell subtypes, such as Tfh cells and resting memory T cells. With TCGA LUSC RNA-seq expression data and LM22 files being input files, CIBERSORT was implemented in “relative mode” to estimate the relative abundance of tumor infiltrating immune cells.

**Figure 1:**
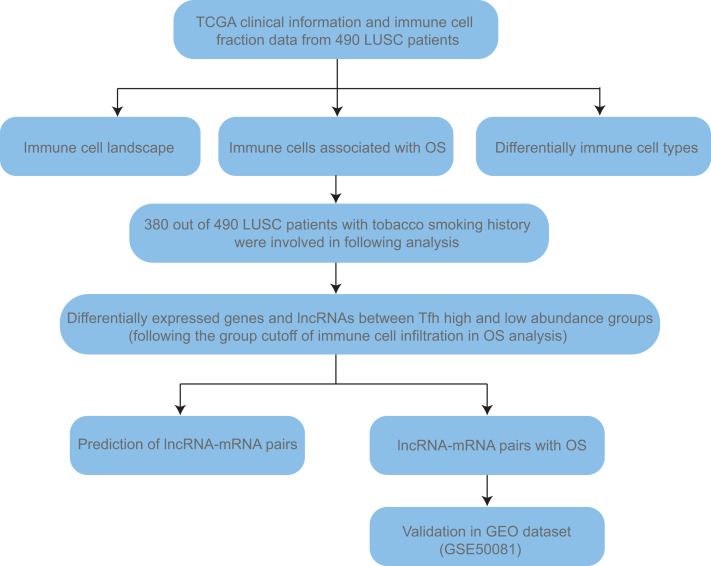
The workflow of this study.

Secondly, the clinical information, including age, gender, TNM stage, and smoking history (Table S1), together with TCGA RSEM normalized RNA-seq gene expression data (Illumina HiSeq) of 490 LUSC patients were obtained from UCSC Xena (https://xena.ucsc.edu/public). TNM stage is a kind of cancer staging system developed by the American Joint Committee on Cancer. T, N, and M mean tumor, node and metastasis, respectively. Lifelong non-smokers (*n* = 17) means person who was not smoking at the time of the interview and has smoked less than 100 cigarettes in their life. Current reformed smokers were defined as people who was not smoking at the time of the interview but has smoked at least 100 cigarettes in their life. According to the duration of cessation, they were classified into current reformed smokers for >15 years (*n* = 78) and ≤ 15 years (*n* = 247). The number of current smokers was 132. Given that people haven’t smoked for 15 years could discontinue lung cancer screening (Moyer & Force, 2014) and the sample size of non-smokers was too small, we combined non-smokers and current reformed smokers for >15 years as “non- + ex-smokers” group to do the comparison. Finally, the validation cohort (GSE50081, Affymetrix HG-U133_Plus_2) was downloaded from GEO (Gene Expression Omnibus, https://www.ncbi.nlm.nih.gov/gds/) to confirm the prognosis value of certain key signature. Detailed workflow was shown in Fig. 1.

### Overall survival (OS) analysis

On the basis of immune cell fraction, LUSC patients were divided into two groups. Combined with patients’ survival data, tumor infiltrating immune cells that had prognosis value were screened out. OS analysis was performed via cox proportional hazards regression model by R language with survminer package, and visualized by survival package. After inputting the survival time and the end point information (means patient dead or alive), Kaplan–Meier survival analysis and log-rank tests were used to obtain the survival curve. In OS analysis, the cutoff of high and low group was set by the optimal *P* value according to the infiltration abundance of each patient.

### Differentially expressed genes (DEGs) and lncRNAs (DElncRNAs)

380 out of 490 LUSC patients with smoking history were involved in this analysis. We continued to use the same cutoff with OS analysis to divide high and low group following the infiltration abundance estimated by CIBERSORT of each patient. According to immune cell fraction, patients were classified into high and low expression groups. The fold change expression of each gene in high and low groups was calculated and log2-transformed. The DEGs and DElncRNAs between two groups were screened with the threshold of —log2 (fold change)— > 1 and adjusted *P* value < 0.05 (*P* value was adjusted by FDR method). The OS analysis of DEGs and DElncRNAs was carried out using similar methods as described in OS analysis section.

### Prediction of lncRNA-mRNA pair and ceRNA network construction

Based on the targeted miRNA dataset of lncRNA or mRNA downloaded from the miRanda (http://www.microrna.org/microrna/home.do), DEGs and DElncRNA target miRNAs were found. After inputting a two-column file including the information of DElncRNAs and its target miRNAs, Cytoscape (venison 3.6.1) would exhibit a ceRNA network.

### Statistical analysis

All statistical analysis in this study was performed using R language. Two group tests were carried out with un-paired *t*-test or Wilcoxon test, and multiple group comparison was carried out with kruskal-wallis method.

### Result

### The correlation between immune cell infiltration and clinical characteristics of LUSC patients

**Figure 2:**
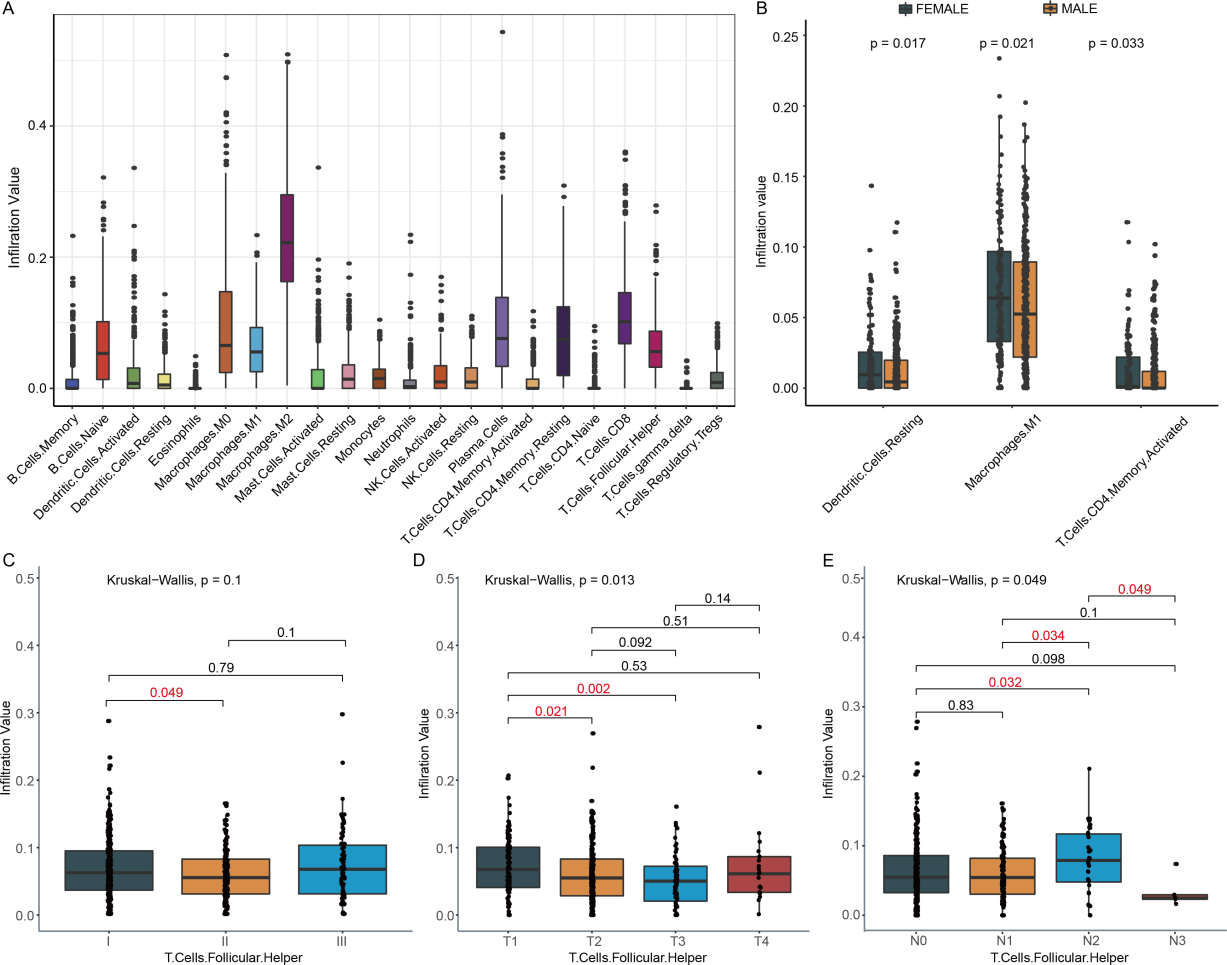
Immune cell infiltration and clinical characters of LUSC patients. (A) Twenty-two immune cells infiltration landscape in LUSC patients was observed. (B) This boxplot showed the distribution of three immune cells in females and males. (C) Tfh cell infiltration in different TNM stages was exhibited. (D) In the T2 and T3 stage, Tfh cells had lower infiltration, compared to the T1 stage. T, tumor (E) In the N2 stage, Tfh cells infiltration had significantly increased, while in the N3 stage, the trends turned into the opposite. N, regional lymph nodes.

We investigated the relationship between the change of immune cell infiltration and clinical characteristics, such as TNM stage, gender and tobacco smoking history. Firstly, the abundance of 22 tumor infiltrating immune cells in 490 patients was analyzed by CIBERSORT. The abundances of M2 Macrophages, CD8 T cells, plasma cells, resting memory CD4 T cells, and Tfh cells in LUSC tumor were higher than that of most other immune cells (Fig. 2A). Secondly, the status of tumor infiltrating immune cell was compared between female and male in this cohort. Compared to male LUSC patients, female LUSC patients had more M1 macrophages, resting dendritic cells, and activated memory T cells CD4 in the tumor (*P* < 0.05, Fig. 2B). Furthermore, the immune cell infiltration at various stages was also analyzed, and only Tfh cell infiltration exhibited statistical difference at various TNM stage. Compared to TNM stage I, Tfh cell infiltration level decreased at TNM stage II (*P* = 0.049, Fig. 2C), while the infiltration of Tfh cell slightly increased at TNM stage III (Fig. 2C). Tfh cell infiltration in detailed TNM stage was compared for further exploration. With tumor growing, the infiltration of Tfh cells declined in T2 and T3 stage, compared to T1 stage (*P* = 0.021 and 0.002, Fig. 2D). However, in T4 stage, the abundance of Tfh cells showed a slight increase (Fig. 2D). In different degrees of lymphatic node metastasis, Tfh cells fraction in N2 stage exhibited significantly rising, compared with N0 stage (*P* = 0.032, Fig. 2E). Moreover, there were other immune cell infiltrations showing significant differences in detailed TNM stage. For example, Macrophages M0 infiltration increased along with the rising of tumor size in T1, T2, and T3, but not in T4 (Fig. S1A). The infiltration of activated mast cells and neutrophils was higher in T2, while the infiltration of activated NK cells presented opposed trend in T2, compared to T1 stage (Fig. S1B & Figs. 2D & 2E). Meanwhile, neutrophils also showed higher infiltration in M1 (Fig. S1). The infiltration of resting mast cells and CD8 T cells declined in T3 (Fig. S1C & Fig. 2F). The infiltration of M1 macrophages and T cells CD8 significantly increased in N1 and N2 stage, respectively, compared with N0 stage (Figs. S1G–S1H).

### DEGs, DElncRNAs and construction of ceRNA network

**Figure 3:**
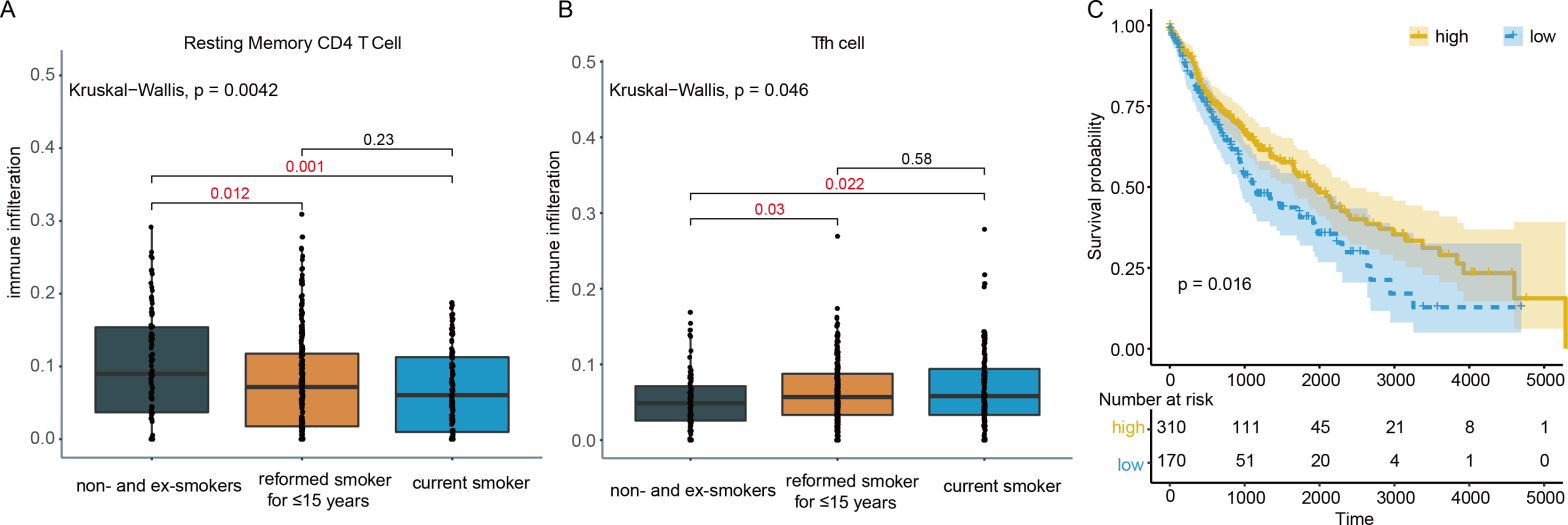
Immune cell infiltration in smoking and non-smoking LUSC patients. (A) Resting memory CD4 T cells showed lower infiltration in current reformed smokers for ≤ 15 years and current smokers. (B) In current reformed smokers for ≤ 15 years and current smokers, Tfh cell infiltration increased significantly. (C) Higher Tfh cell infiltration patients had better OS.

In order to find immune cells that might be affected by tobacco smoking in LUSC, the abundance of 22 tumor infiltrating immune cells were assessed by CIBERSORT. Two types of immune cells exhibited significant difference among non- and ex-smokers, current reformed smokers for ≤ 15 years and current smokers. Resting memory CD4 T cell infiltration in patients not smoking for ≤ 15 years (*P* = 0.012) and current smokers decreased (*P* = 0.001, Fig. 3A), while Tfh cell infiltration in the two groups both increased (*P* = 0.03, 0.022, Fig. 3B), compared to non- + ex-smokers. However, only Tfh cells fraction was related to OS with *P* = 0.016 and hazard ratio (HR) = 0.71 (Fig. 3C). Hence, in the following investigation, 380 LUSC patients with smoking history were divided into high and low group according to the infiltration of Tfh cells to identify functional genes that related to clinical outcomes.

**Figure 4:**
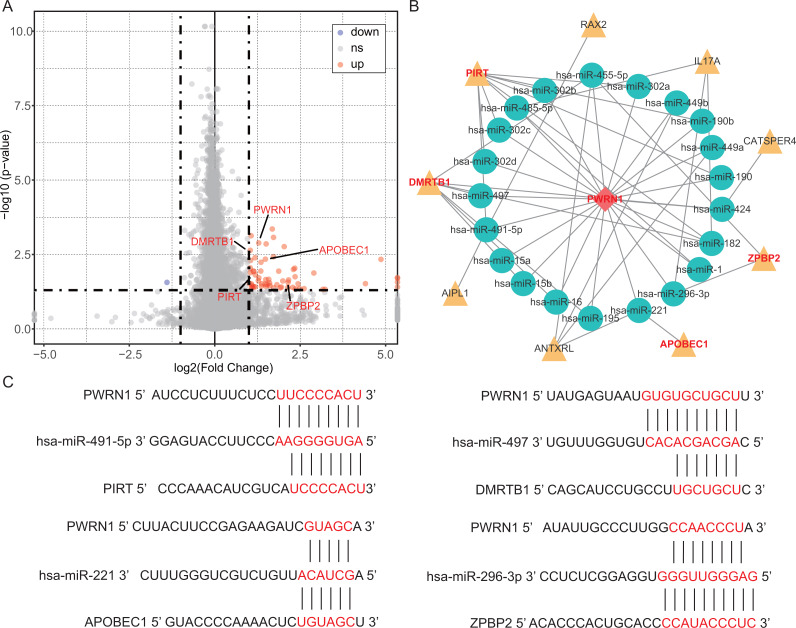
DEGs, DElncRNAs and construction of ceRNA network. (A) The volcano plot showed the DEGs and DElncRNAs. The red point indicated DEGs or DElncRNAs were up-regulated in high Tfh cells infiltration group, while the blue point implied they were down-regulated. ns, not significant. (B) The ceRNA network was built via Cytoscape. Yellow triangle, up-regulated genes. (C) The binding site of lncRNA-miRNA-mRNA was predicted by miRanda.

In the OS analysis, the cutoff threshold of high and low group was set by the best *P* value. And in the analysis of DEGs and DElncRNAs, the group cutoff value was set in accordance with that. From differentially expression analysis, 61 DEGs and 2 DElncRNAs were screened out with the threshold of —log2 (fold change) > 1— and adjusted *P* < 0.05 and showed in the volcano plot (Fig. 4A). The prediction of lncRNA-miRNA and miRNA-mRNA functional pairs were both carried out on miRanda. A ceRNA network centered on DElncRNA PWRN1 was built and visualized in Fig. 4B. The red rhombus presented up-regulated DElncRNA PWRN1, and the yellow triangle pointed to up-regulated DEGs. Blue circular was on behalf of miRNA that PWRN1 and DEGs could both combine. In Fig. 4C, the binding site of partial paired DElncRNA-miRNA-DEGs was shown and all of them had a mirSVR score <−0.1 and PhastCons score >0.5. mirSVR and PhastCons score indicate thermodynamic stability and conservation, respectively. Lower mirSVR score presents better binding stability and higher PhastCons score means more conservative

### OS analysis and validation

**Figure 5:**
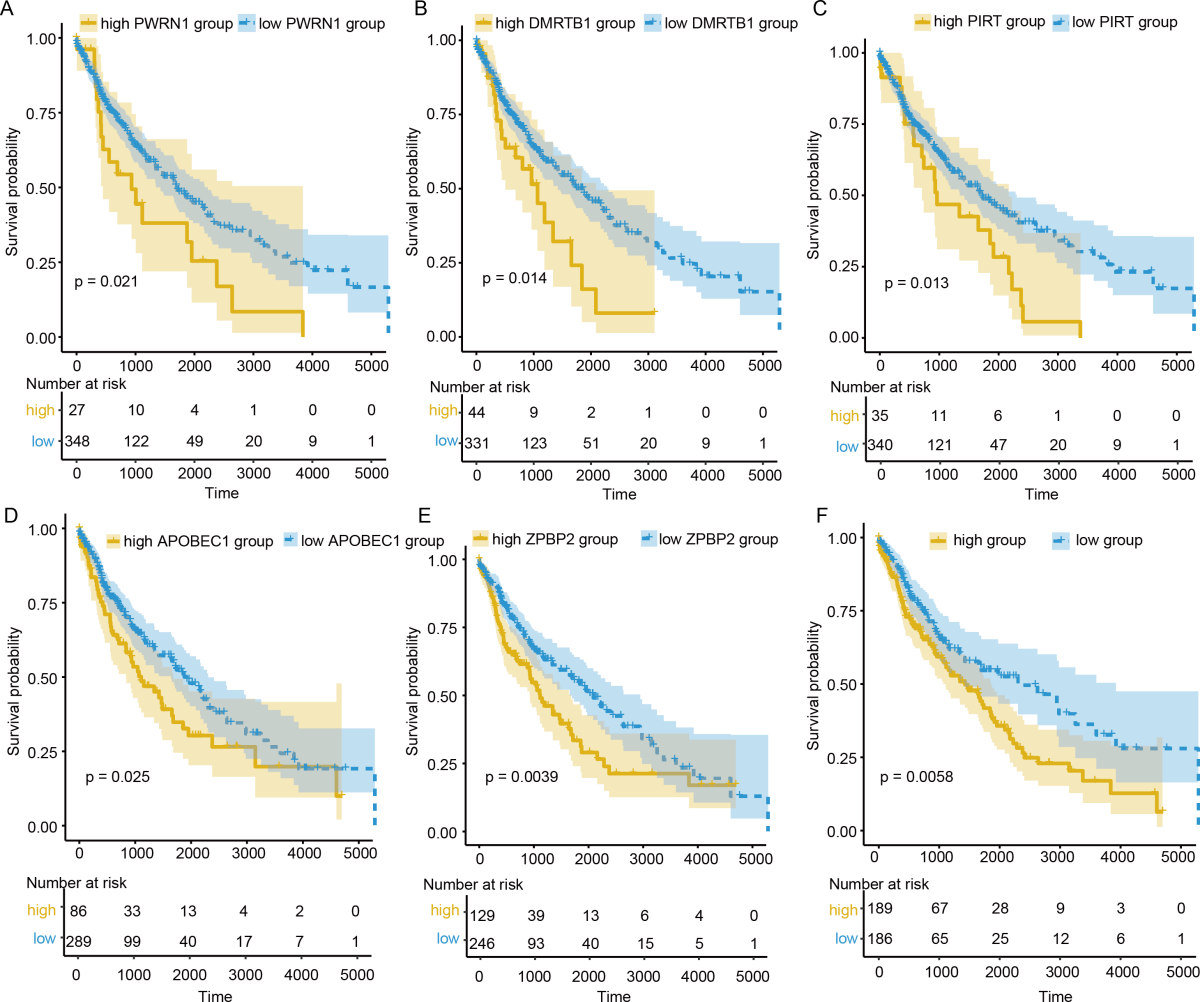
OS analysis of DElncRNAs, DEGs and signature. (A–E) The patients with low expression PWRN1 (A), DMRTB1 (B), PIRT (C), APOBEC1 (D) and ZPBP2 (E), had better OS. (F) Low signature group showed longer survival time.

**Figure 6:**
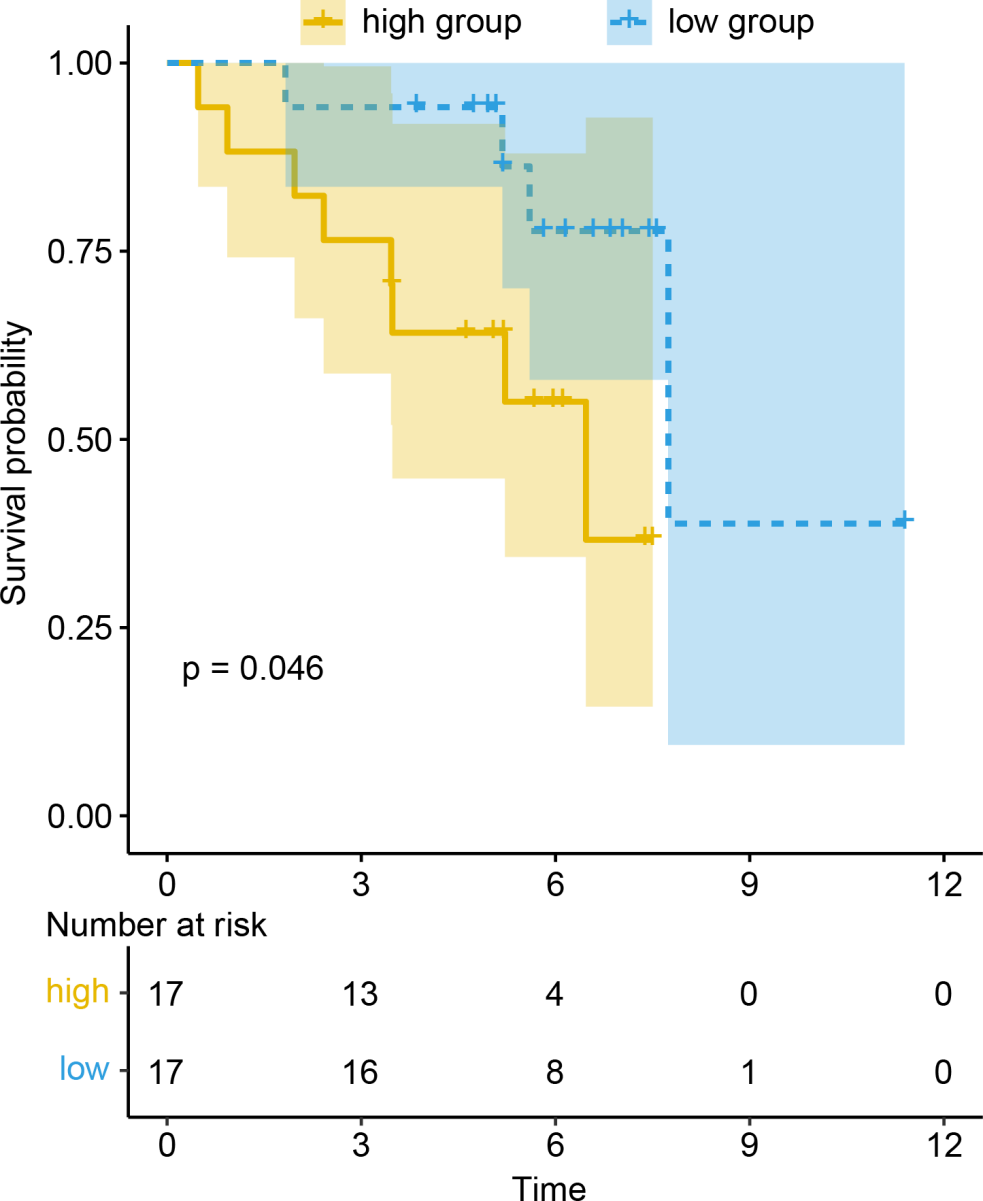
Prognosis value of signature was confirmed in the GEO dataset.

To evaluate the prognostic value of DEGs and PWRN1 in ceRNA network, OS analysis was performed. Comparing to high expression group, the group with low expression of PWRN1 (*P* = 0.021), DMRTB1 (*P* = 0.014), PIRT (*P* = 0.013), APOBEC1 (*P* = 0.025), and ZPBP2 (*P* = 0.0039) had better OS (Figs. 5A–5E). HR value of high PWRN1, DMRTB1, PIRT, APOBEC1, and ZPBP2 expression group was 1.74, 1.75, 1.72, 1.58, 1.46, and 1.55 respectively (Table 1). The 95% CI of them was also exhibited in Table 1. The cutoff value of high and low expression in this OS analysis was also set by the best *P* value. When we averaged the expression of PWRN1, DMRTB1, PIRT, APOBEC1, and ZPBP2 to make them as a signature and divided the patients by median value of signature expression, low expression group showed better OS than high expression group (Fig. 5F, *P* = 0.0058, HR = 1.55). Subsequently, the finding was validated in GSE50081. The above-mentioned five genes were combined and 34 LUSC patients with smoking history were grouped in the same way. The low signature expression group also demonstrated better prognosis clinical outcomes with a *P* value of 0.046 (Fig. 6) and a HR score of 3.57 (Table 1).

## Discussion

NSCLC is the most common kind of lung cancer (accounting for 83% of cases) with a poor 5-year survival of less than 20%. LUSC, a subtype of NSCLC, associated closely to tobacco smoking. Cigarette smoking exposure could not only induce genetic alteration, but also suppress the immune system so that to contribute to carcinogenesis and metastasis. Previous studies found that the infiltration of some immune cells, such as Tfh cells, was associated with better OS. Therefore, in this study, we tried to explore the reason why higher infiltration of Tfh cells corresponds to better clinical outcomes from the perspective of ceRNA.

Firstly, the immune cell infiltrations in LUSC patients were observed. And we found that M2 Macrophage, CD8 T cells, plasma cells, resting memory CD4 T cells, and Tfh cells had higher infiltration compared to other immune cells. In NSCLC, M2 macrophage promotes epithelial to mesenchymal transition (EMT) and causes malignancy (Guo et al., 2019). Higher infiltration of CD8 T cells, plasma cells and Tfh cells was associated with better OS of patients with cancer (Knief et al., 2016; Miotto et al., 2010; Zhu & Zhang, 2019). Subsequently, we analyzed the infiltration of immune cells in different TNM stage, and only Tfh cells presented significant differences in various TNM stages. Compared to stage I, Tfh cell exhibited a slight decline in stage II, but in stage III, the trend was not going on, which was partly in agreement with previous report (Guo et al., 2017). In the detailed TNM stage, Figs. 2D and 2E actually showed negative correlation to T and positive association to N stage. There are two possible explanations about the decline in stage II. First, for the immune infiltration abundance of each cell estimated by CIBERSORE is relative value, the decline of Tfh cell infiltration abundance in stage II may result from the increase or decrease of other immune cells. Second, in different stages of NSCLC, the change of Tfh subtypes is inconsistent. The Tfh1 cell abundance in stage II-IV decreased, while Tfh2 and Tfh17 abundances in stage II-IV increased (Qiu et al., 2018) and the ratio of Tfh1, Tfh2, and Tfh17 abundances might lead to the Tfh cell infiltration abundance changing. Further, benefit from immune escape, tumor cells can migrate to other space. CD4+CXCR5+ T cell population was examined to define Tfh cells (Ma et al., 2016) and PD-1+ CD4+ CXCR5+ T cells presented lower capacity to assist CD8 T cell (Ma et al., 2016). Hence, we supposed that this Tfh cell subtypes might be chosen higher infiltration so that the tumor stage and metastasis could progress. The decrease of Tfh cell abundance in N3 stage might because the sample size of N3 stage patients was too small to make result not reliable (the patient number in N1, N2, and N3 stage was 304, 128, 36, and 5). Further, previous reports showed that female patients with NSCLC had better OS (Freudenstein et al., 2020). In our results, we found some clues to explain it. The dendritic cells (Freudenstein et al., 2020), M1 macrophages (Cornelissen et al., 2014), and activated CD4 T memory cells (Zhang et al., 2020) that exhibited anti-tumor capacities were all demonstrated higher infiltration in females, compared to the male. That might because the different levels of estrogen and androgen in females and males. The differential effect of estrogen on immune function reflects not only the concentration of estrogen, but also reflects the density, distribution and type of estrogen in immune cells (Klein & Flanagan, 2016). Estrogen receptor (ERs) are expressed in various immune cells, such as macrophages, T cells and dendritic cells (Phiel et al., 2005). Estrogen could influence functional activity of innate immune cells and adaptive immune responses. Androgens exhibited higher concentrations in post-pubertal males than females could inhibit immune cell activity (Roberts, Walker & Alexander, 2001).

**Table 1:** Hazard Ratio and *P* value of each group.

**Source**	**Group**	**Hazard Ratio (HR)**	**logrankP**	**lower 95% CI**	**upper 95% CI**
TCGA	High PWRN1 group	1.74	0.02	1.08	2.81
TCGA	High DMRTB1 group	1.75	0.01	1.11	2.76
TCGA	High PIRT group	1.72	0.01	1.11	2.66
TCGA	High ZPBP2 group	1.58	0.004	1.15	2.16
TCGA	High APOBEC1 group	1.46	0.026	1.05	2.05
TCGA	High signature group	1.55	0.006	1.13	2.12
GSE50081	High signature group	3.57	0.046	0.94	13.59

Given that tobacco smoking exposure was a vital factor of LUSC carcinogenesis, the immune cell infiltration in LUSC current reformed smokers, current smokers and non-smoker patients was analyzed. The infiltration of Tfh cells increased in smokers, while that of resting memory CD4 T cells decreased. Tobacco smoking exposure could result in CD4+ memory T cells switching from resting to activation (Li et al., 2018). Further, cigarette smoking could also trigger the activation of dendritic cells to facilitate CD4+ T cells differentiation into Tfh cells (Qiu et al., 2017; Ramiscal & Vinuesa, 2013). That might be the explanation of increased infiltration of Tfh cells and decreased infiltration of resting memory CD4 T cells. After cox proportional hazards regression analysis, patients with high infiltration of Tfh cells showed better clinical outcomes, which was also consistent with previous findings (Ma et al., 2016; Xu et al., 2020).

To find potential molecule mechanism between Tfh cells and OS, LUSC patients with smoking history were divided into two groups according to the infiltration of Tfh cells so that DElncRNAs and DEGs could be screened out. It was found that the combination of DElncRNA PWRN1, DEG DMRTB1, PIRT, APOBEC1, and ZPBP2 was significantly related to OS. However, the function of these genes have not been studied adequately in LUSC. Prader-Willi region non-protein coding RNA 1(PWRN1) located in chromosome 15 and up-regulated PWRN1 suppressed cell proliferation and tumor growth in gastric cancer (Chen et al., 2018). But in our study, high expression PWRN1 was related to worse clinical outcomes, which might be because PWRN1 was a strongly tissue-specific lncRNA so that PWRN1 could both act as proliferation-promoting and tumor suppressor role. For example, NEUROD1/2 functions as growth-promoting gene in HPNE cells, but as suppressor of proliferation in HMEC cells (Sack et al., 2018). DMRT like family B with proline rich C-terminal 1 (DMRTB1) mutation might relate to human colorectal cancer liver metastasis (Ma et al., 2018). Ahmadreza Niavarani et al. found that strong expression of apolipoprotein B mRNA editing enzyme catalytic subunit 1 (APOBEC1) was associated with short survival time in pan-cancer, which was in line with our result (Niavarani et al., 2018). And that might because APOBEC1 behave as a consistently hypo-methylated gene in pan-cancer (Niavarani et al., 2018). Wiemels JL et al. identified new risk loci, located on 17q12 near Zona pellucida binding protein 2 (ZPBP2), could regulate gene expression by local interactions with ZPBP2, impacting function of hematopoietic and growth-regulation pathways in childhood acute lymphoblastic leukemia (Wiemels et al., 2018). The DElncRNA or DEGs in PWRN1-mediated ceRNA network was related to clinical outcomes or cancer development somehow. Therefore, I reckoned that the PWRN1-mediated ceRNA network was a potential prognostic biomarker in LUSC.

## Conclusions

In conclusion, it was found that Tfh cells presented higher infiltration in LUSC current reformed smokers for ≤ 15 years and current smokers, while resting memory CD4 T cells had lower infiltration. Tfh cells infiltration was not only associated with better OS, but also varied from different degrees of the TNM stage. The signature consisting of PWRN1 and its predicted targeted mRNAs was dysregulated in different degrees of Tfh cell infiltration and might indicate patients’ OS. The prognostic value of the signature was also confirmed in GEO dataset (GSE50081).

##  Supplemental Information

10.7717/peerj.9996/supp-1 Supplemental Information 1 The boxplot exhibited the various immune cells in different T stage (tumor), N stage (regional lymph nodes) or M stage (distant metastases)

10.7717/peerj.9996/supp-2 Supplemental Information 2 The immune cell infiltration abundance estimated by CIBERSORT and clinical information of each patient

## Additional Information and Declarations

### Microarray Data Deposition

The following information was supplied regarding the deposition of microarray data: Data is available at GEO: GSE50081.

### Data Availability

The following information was supplied regarding data availability: Data is available at NCBI GEO: GSE50081 and at Xena: TCGA.LUSC.sampleMap/HiSeqV2 (https://xenabrowser.net/datapages/?dataset=TCGA.LUSC.sampleMap%2FHiSeqV2&host=https%3A%2F%2Ftcga.xenahubs.net&removeHub=https%3A%2F%2Fxena.treehouse.gi.ucsc.edu%3A443).
