# Supplementary figures and images for: Immune cell landscape analysis reveals prognostic immune cells and its potential mechanism in squamous cell lung carcinoma
Source: PeerJ. 2020 Oct 5;8:e9996. doi: 10.7717/peerj.9996 (PMC7543728; doi:10.7717/peerj.9996)

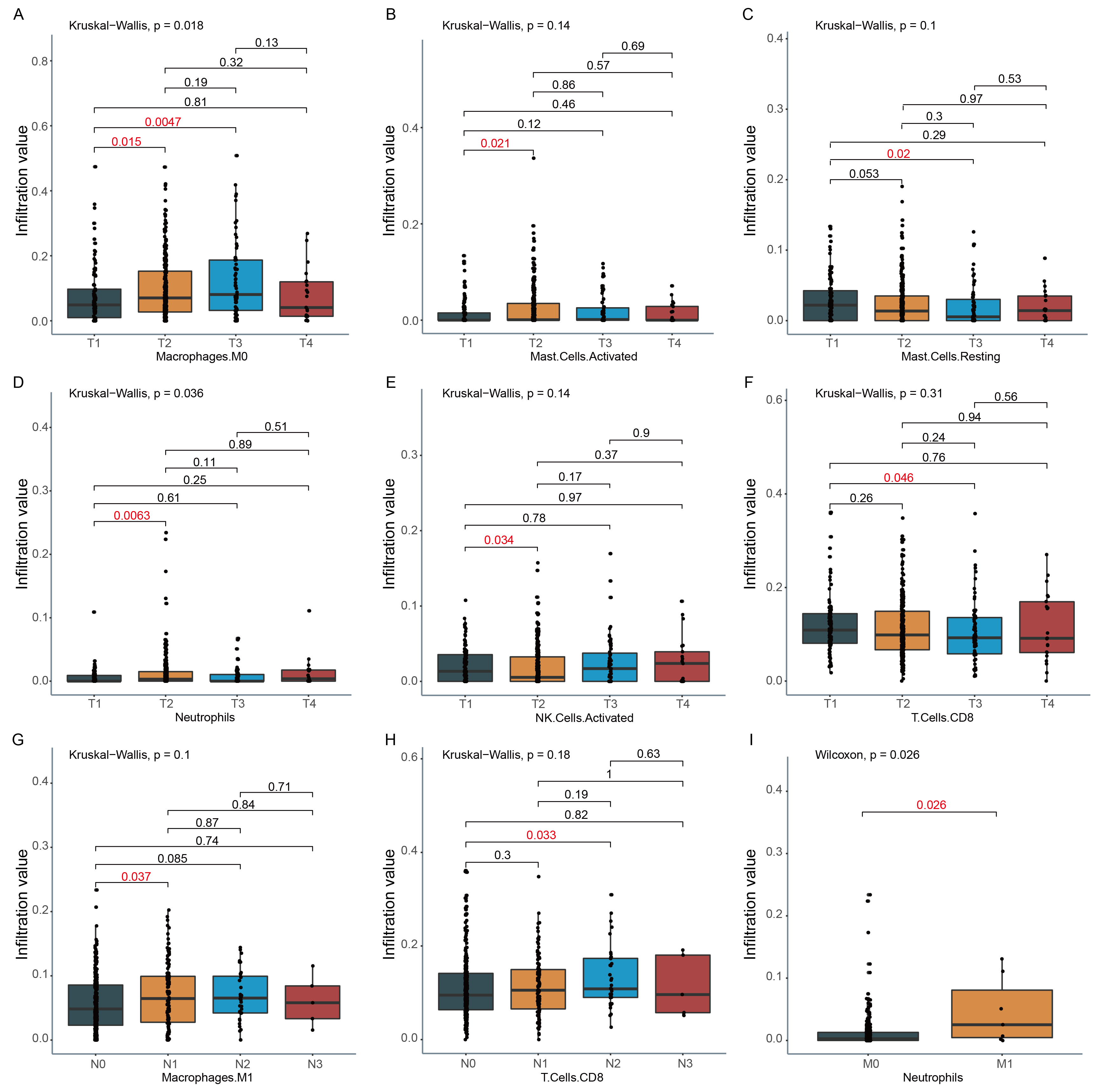

Supplement: Supplemental Information 1 [file peerj-08-9996-s001.png]
